# Regulation of opioid receptor signalling: Implications for the development of analgesic tolerance

**DOI:** 10.1186/1756-6606-4-25

**Published:** 2011-06-13

**Authors:** Karim Nagi, Graciela Piñeyro

**Affiliations:** 1 Département de Pharmacologie, Faculté de Médecine, Université de Montréal, Canada; 2 Département de Psychiatrie, Faculté de Médecine, Université de Montréal, Montréal, Québec, H3C 3J7, Canada; 3 Centre de recherche du CHU Sainte-Justine, 3175, Côte-Sainte-Catherine Montréal, Québec, H3T 1C5, Canada

## Abstract

Opiate drugs are the most effective analgesics available but their clinical use is restricted by severe side effects. Some of these undesired actions appear after repeated administration and are related to adaptive changes directed at counteracting the consequences of sustained opioid receptor activation. Here we will discuss adaptations that contribute to the development of tolerance. The focus of the first part of the review is set on molecular mechanisms involved in the regulation of opioid receptor signalling in heterologous expression systems and neurons. In the second part we assess how adaptations that take place *in vivo *may contribute to analgesic tolerance developed during repeated opioid administration.

## I. Introduction

Opiates are among the most effective analgesics known but their clinical use is limited by severe side effects. Some of these undesired actions including tolerance, dependence and abuse usually appear after repeated opioid administration, and have been linked to adaptations that take place in order to counteract prolonged opioid receptor activation [1,2]. Adaptive changes have been described at different organizational levels within the central nervous system, ranging from receptor and cellular alterations to functional modifications of different neuronal networks [3,4]. Regulation that occurs at the receptor level results in the progressive waning of signalling efficacy and is known as desensitization. Mechanisms of opioid receptor desensitization were initially characterized in immortalized cell lines [5] but more recent studies have extended observations to cultured neurons [6-8] and animal models [9-12]. Here we will review these findings with special focus on recent efforts to understand how regulation of receptor signalling may contribute to analgesic tolerance developed during repeated opioid administration.

## II. Opioid receptor regulation in heterologous expression systems

### Opioid receptor desensitization and endocytosis

Studies in immortalized cell lines have shown that like for many other G protein-coupled receptors (GPCRs), opioid receptor activation involves a series of conformational changes [13,14] that trigger signalling and regulation. Regulatory steps usually start with phosphorylation of the receptor [15,16] followed by βarrestin recruitment [17,18] and disruption of receptor signaling via G-protein coupled effectors [19,20]. In addition, since arrestins bind to the coat structure of clathrin-coated pits [21,22] a great majority of ligands that promote functional desensitization also enhance sequestration. The frequent association of these two processes was initially taken as an indication that opioid receptor internalization and desensitization were causally linked [23,24], an interpretation that was reinforced by studies showing that morphine failed to induce both, internalization [25,26] and desensitization [27,28]. Moreover, given that morphine induces more analgesic tolerance than agonists capable of triggering a full regulatory response [29-31], its high potential for tolerance was initially considered as the consequence of cellular adaptations to counteract sustained signaling by receptors that were unable to desensitize or internalize [23,25,27]. However, morphine's failure to trigger regulation of receptor signaling cannot be extended to all systems since several reports have shown that this drug causes βarrestine recruitment, desensitization and endocytosis of mu (MORs) [32-35] and delta (DORs) [35,36] opioid receptors. Moreover, animal studies have confirmed that receptor regulation is essential for morphine tolerance to develop since transgenic mice lacking βarrestin2 display enhanced, longer lasting analgesic responses to the drug [37,38]. Based on these observations, the mechanism of morphine tolerance was reconsidered and the contribution of endocytosis re-evaluated. This alternative hypothesis proposes βarrestin-driven receptor-G protein uncoupling (desensitization) as the mechanism responsible for the loss of morphine's analgesic action, which is in turn exacerbated by receptor failure to internalize and undergo resensitization [38,39]. However, this mechanism cannot be generalized to agonists that induce internalization or even to other opioid ligands that like morphine fail to do so. In particular, homologous desensitization does not account for tolerance induced by AR-M1000390, a low internalizing DOR agonist [40] whose repeated administration induces tolerance without modifying receptor ability to activate the G protein [12]. Moreover, internalization *per se *warrants neither resensitization nor absence of tolerance. For example although SNC-80 produces rapid internalization of DORs [41], its systemic administration induces long lasting analgesic tolerance after a single administration [11]. MOR activation by efficiently internalizing ligands is also associated with progressive loss of analgesic efficacy, although unlike SNC-80, MOR agonists require repeated administration for tolerance to develop [42].

### Post-endocytic sorting of opioid receptors

Functional consequences of receptor internalization cannot be fully understood without considering what happens after sequestration. Hence, if internalization is associated to receptor recycling, the process allows to restore functional receptors to the membrane [43,44]. Both MORs [39] and DORs [41,45,46] have been shown to undergo recycling that contributes to their functional resensitization. In contrast, if the receptor is preferentially directed towards the lysosomal compartment, internalization leads to prolonged desensitization due to its proteolytic degradation [47,48].

**Figure 1:**
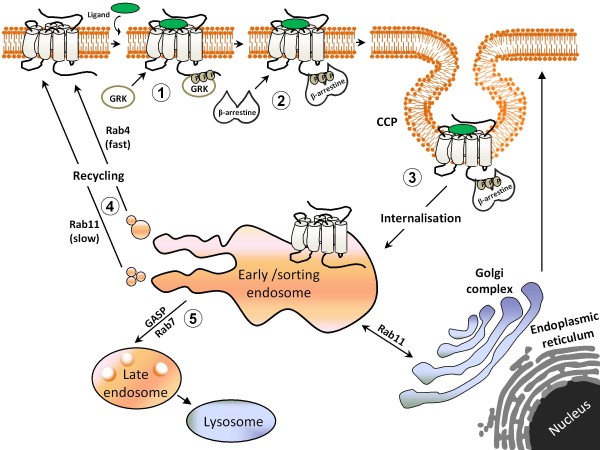
**Steps involved in the homologous desensitization of GPCRs**. According to the classical model of homologous desensitization, receptor activation by an agonist induces a series of conformational changes that trigger receptor signalling and regulation. The first of these regulatory steps is receptor phosphorylation by GRK (1). Once phosphorylated receptor affinity for βarrestin increases, enhancing interaction between the two proteins (2) and promoting internalization (3). Internalized receptors are then directed to early/sorting endodomes where interaction with different regulatory proteins will allow them to recycle back to the membrane (4) or will directed towards degradation (5).

Factors responsible for sorting opioid receptors to these alternative pathways are multiple, and some are specific to each receptor subtype. An important determinant of lysosomal sorting is ubiquitination [49] and both DORs and MORs have been shown to become ubiquitinated and degraded after stimulation. However, while DORs are ubiquitinated within minutes of activation [48,50] MORs require various hours of stimulation [51]. On the other hand, despite their rapid ubiquitination, DORs are not immediately degraded but may remain withheld in the endosomal compartment [52] for periods that may last as long as four hours of agonist exposure [11,53]. The discrepancy in the time required to undergo ubiquitination and degradation is consistent with the fact that DOR sorting towards the degradation path is not dependent upon ubiquitin addition [48,54]. Instead, their trafficking to the late endosomal compartment relies, at least in part, upon interaction with sorting proteins of the GASP (G protein coupled receptor associated sorting protein) family [47,54,55]. MORs also bind GASPs, but the low affinity of this interaction seems to account for their lower tendency to undergo lysosomal targeting and degradation as compared to DORs [47,55]. In addition, a primary sequence within the C-terminal domain of some MOR isoforms facilitates their active targeting towards the recycling pathway [56]. Yet this is not the only determinant of MOR recycling since isoforms lacking the sorting sequence may also be sent back to the membrane after internalization [57,58]. Indeed, MORs are also known to constitutively interact with neuronal membrane glycoprotein M6a, which accelerates their recycling after internalization [59]. In addition, MOR and DOR recycling may be dynamically regulated through receptor phosphorylation [46,60] and interactions with βarrestin1 and βarrestin2 [61]. See Figure 1 for a schematic representation of the process of homologous desensitization.

## III. Regulation of opioid-mediated responses in neurons

Studies in slices and neuronal cultures have confirmed that neuronal MORs may undergo βarrestin-dependent internalization by full agonists like DAMGO and partial agonists like morphine [6]. Importantly, internalization following short term exposure to morphine varies across different neuronal populations. In particular, while this drug induced MOR endocytosis in striatal neurons [6] it was without effect in those of the locus coeruleus (LC) [7,62], dorsal root ganglion (DRG) [8] or enteric plexus [63,64]. Such differences are not surprising since as a partial agonist internalization by morphine is expected to be influenced by the level and type of endocytic proteins expressed in each neuronal subtype. In keeping with this interpretation prolonged morphine exposure was found to enhance dynamin expression in enteric neurons, turning the agonist into an internalizing ligand [64].

Functional assays have revealed that receptor stimulation by morphine and by more efficacious ligands are all capable of inducing functional desensitization of MOR-mediated neural responses. However, the relationship between internalization and loss of signaling capacity seemed influenced by the cell type and effector considered. In particular, although short-term (20-30 min) exposure of DRG and LC neurons to internalizing ligands was associated with desensitization of channel-mediated responses, sequestration was not necessary for desensitization to take place [7,62]. On the other hand, interfering with sequestration of striatal MORs resulted in partial reduction of the desensitization of cyclase responses evoked by DAMGO and morphine [65]. Apart from distinct need for internalization, the mechanistic basis of desensitization seems also effector specific. In particular, N-type Ca^+2 ^channels in DRG neurons were shown to undergo rapid desensitization that was not affected by βarrestin2 knockout [66] but was instead mediated by a heterologous mechanism acting downstream of the receptor [8,67,68]. In contrast, desensitization of G protein activated inward rectifier K+ (GIRK) channels in LC neurons was homologous [62], and dependent on the combined activity of ERK1/2, G protein receptor kinase 2 (GRK2) and arrestin2 [68]. βarrestin2 was also involved in the desensitization of cyclase responses following sustained exposure of striatal MORs to morphine and DAMGO [65]. However, in spite of its ability to trigger MOR regulation, exposure to morphine induced superactivation of the striatal cAMP cascade [69], suggesting that MOR desensitization when associated to this effector might not be enough to completely avoid cellular compensatory mechanisms.

Information concerning post-endocytic sorting of neuronal opioid receptors is quite limited. Studies in DRG neurons indicate that MORs undergo constitutive recycling which requires βarrestin2-dependent internalization and trafficking through a monensin sensitive compartment [8,66]. Agonist stimulation of these receptors induces their colocalization with Rab4 and Rab11, indicating redistribution of DRG MORs to recycling endosomes [8]. Functional consequences of receptor recycling have been assessed in LC neurons. In these cells MOR desensitization by the endogenous agonist Met-enkephalin could be reversed upon agonist removal, resulting in complete recovery of receptor ability to evoke GIRK channel activation [7,70]. Interestingly, the mechanism involved in resensitization was different depending on whether desensitization was accompanied or not by internalization. Indeed, when recovery took place after internalization, resensitization was sensitive to recycling disruption by monensin [70]. In contrast, when internalization was blocked, desensitization and recovery could both take place at the membrane [7], pointing to the existence of multiple, complementary mechanisms for achieving similar regulatory control of opioid receptor signaling. The existence of multiple, complementary and cell-specific regulatory responses were not necessarily anticipated from studies in heterologous systems. They should nonetheless be carefully considered since they may point to the impossibility of developing a single, universal strategy for avoiding analgesic tolerance.

## IV. In vivo regulation of opioid receptor signaling

A critical question in understanding long term effects of opioids is whether regulatory responses described in cellular models are also triggered *in vivo*, and if so, what are their behavioral correlates. Insight into these issues has been obtained by assessing regulatory responses triggered by the release of endogenous opioids or following exogenous administration of different opioid receptor agonists.

### Opioid receptor regulation by release of endogenous opioids

The release of endogenous opioid peptides during noxious stimulation may produce phosphorylation [10,71] and internalization [72,73] of central and peripheral MORs. These regulatory responses were triggered by stimuli that lead to development of persistent pain syndromes [10,72,73] but not by acute noxious stimulation [74], a difference that has been attributed to higher receptor occupancy in the former than the latter. Consistent with this interpretation, acute painful stimuli may provoke MOR internalization if the stimulation is accompanied by administration of peptidase inhibitors that prevent rapid degradation of the released opioids [75].

From a functional point of view MOR phosphorylation following sciatic nerve ligature was correlated with desensitization of receptor ability to stimulate the G protein, development of cyclase superactivation, appearance of thermal hyperalgesia and reduced analgesic response to exogenous opioids [10,76]. The use of β-endorphin knock-out mice confirmed a causal link between MOR phosphorylation by this peptide and reduced responsiveness to exogenous ligands since sciatic nerve ligature in knock-out animals failed to produce both [10]. On the other hand, regulatory mechanisms triggered by endogenous opioids during the course of chronic inflammatory pain seem to have a protective effect against morphine tolerance. Indeed, depletion of opioids from white blood cells of rats that had received an intra-plantar injection of complete Freund's adjuvant (CFA) prevented MOR sequestration in primary afferents. Together with inhibition of sequestration, opioid depletion was associated with the exacerbation of cyclase superactivation and analgesic tolerance produced by intraplantar administration of morphine [72].

### Opioid receptor regulation by administration of opioid agonists

As mentioned in previous sections, studies characterizing homologous desensitization of opioid receptors have prompted two alternative hypotheses in order to explain morphine tolerance. Although not necessarily compatible at other levels, both conceptualizations agree upon the fact that receptor internalization may have a protective effect against the loss of morphine's analgesic efficacy. This possibility has been directly assessed by Kim et al, 2008 [77] who used a knock-in mouse model in which wild-type MORs were replaced with a mutant receptor capable of undergoing rapid morphine-dependent sequestration [27]. What the authors report is that a 5 day treatment which almost abolished morphine analgesia in wild type mice produced no tolerance in knock-in animals [77]. The idea that MOR internalization negatively influences the development of tolerance is also supported by experiments carried out in wild type animals where the rate at which analgesic efficacy diminishes is faster for low internalizing opiates like morphine or heroin [38,77-79] than for efficiently internalizing agonists like DAMGO [80], etorphine [38,81] or methadone [38,77]. However, the protective effect of internalization is limited, since treatments of 7 days or longer will all eventually induce analgesic tolerance [37,78,81,82] independent of the degree of internalization triggered by the agonist. Although part of this effect may be accounted for by adaptations that take place at synaptic and network levels [4], receptor adaptations are also involved since tolerance is paralleled by receptor desensitization [37,78,81-84] and down regulation [81,85]. Thus, if the intent is to eventually harness opioid receptor regulation as a means of prolonging opioid analgesia, it will be necessary to look beyond internalization. Characterization of the post-endocytic mechanisms whereby *in vivo *sequestration provides transient protection from tolerance as well as a better understanding of the causes leading to down-regulation and delayed loss of analgesic efficacy, seem essential steps for the rational development of novel, longer acting opioid analgesics.

The relationship between endocytic trafficking and analgesia is also being actively pursued for DORs. A report by Pradhan et al, 2009 [11] has recently established that a single injection of SNC-80 produced *in vivo *internalization of DORs which was paralleled by the development of acute analgesic tolerance. In contrast, the administration of an equianalgesic dose of AR-M1000390 produced neither internalization nor modification of subsequent analgesic responses. Based on these observations it would be tempting to speculate that acute tolerance to DOR agonists is determined by internalization. However, this interpretation is ruled out by results obtained with deltorphin II, whose administration is free of acute tolerance [86] despite its high internalization capacity [87]. The reason for the distinct tolerance potential displayed by SNC-80 and deltorphin II remains to be elucidated, but analysis of post-endocytic trafficking could shed some light onto the issue. Studies in immortalized cell lines indicate that internalization by SNC-80 is not followed by for receptor recycling [41] and *in vivo *experiments show that four hours after its systemic administration SNC-80-stimulated DORs remain trapped in the cytosol while analgesic tolerance is maximal [11]. On the other hand, internalization by deltorphin analogues is associated with partial recycling and resensitization of DOR signalling [88], both of which may contribute to a faster recovery of analgesic efficacy upon repeated administration of this type of ligands [86]. Based on these observations it would be interesting to determine whether preferential sorting towards the recycling path is what makes deltorphin II less prone to tolerance than SNC-80. The molecular underpinnings of agonist-specific sorting could include stabilization of agonist-specific conformations that distinctively interact with sorting proteins such as GASPs or βarrestins. This reasoning is supported by reports indicating that DORs may adopt multiple active conformations [14] that are distinctively modulated by Src [89,90], which is in turn involved in the modulation of DOR recycling efficacy [46].

**Figure 2:**
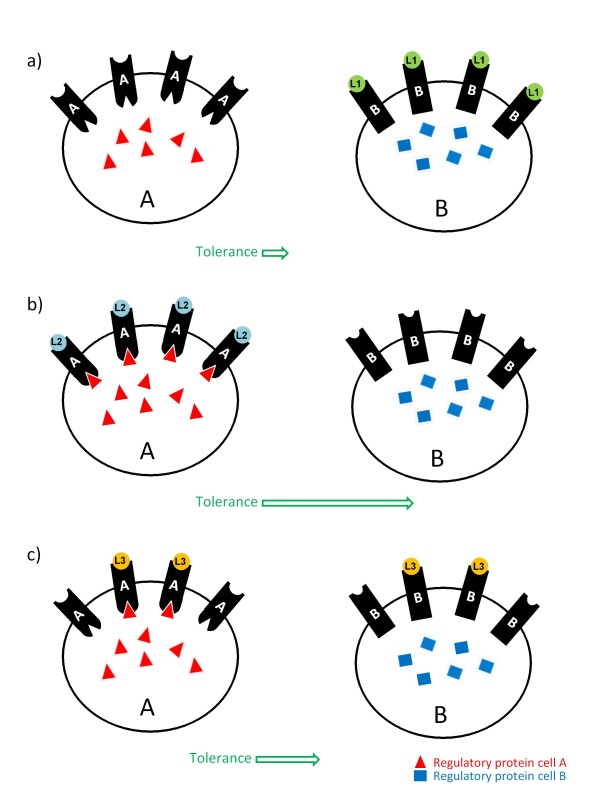
**Biased agonists targeting cell-specific receptor/effector complexes may prove a valid approach for developing longer acting opioid analgesics**. Conformational restrictions within receptor-effector complexes present in *cell A *make them more sensitive to the effect of regulatory proteins than receptor-effector complexes in *cell B*. If these differences make analgesic responses mediated by *cell A *more prone to tolerance than those mediated by cell B, *a) *agonists that preferentially recognize and activate complexes in cell B are expected to produce longer lasting analgesia than *b) *an equally efficacious agonist that preferentially stimulates complexes *in cell A *or *c) *agonists that do not discriminate among complexes.

Comparison of long-term analgesic actions evoked by internalizing and non-internalizing DOR agonists has confirmed that similar to what was observed for MORs, both types of ligands induce tolerance after repeated administration [12,86]. Remarkably, for some agonists desensitization of DOR-mediated signals takes place at the receptor while for others desensitization occurs at the level of the effector. For example, while tolerance by SNC-80 involves receptor desensitization AR-M1000390 leaves DORs unaffected but reduces Ca+2 channel ability to respond to stimuli [12]. An additional level of diversity that has been described in the regulation of DOR-mediated *in vivo *responses is that different behaviours display distinct sensitivity to tolerance. For example, while repeated AR-M1000390 administration did not modify drug ability to induce anxiolytic and psychomotor responses, it induced complete analgesic tolerance [12]. It seems unlikely that "response-specific" tolerance generated by AR-M1000390's is related to its failure to internalize DORs since similar specificity has been described for internalizing ligands. In effect, sustained treatment with SNC-80 led to the progressive reduction of its pro-convulsive but not antidepressant actions [91]. Similarly, sustained administration of deltorphin II resulted in the progressive reduction of antinociceptive but not antihyperalgesic actions induced by this drug [86]. The fact that sustained stimulation of the same receptor may result in different degrees of tolerance for distinct behavioural responses is highly reminiscent of observations obtained in neuronal cultures where signalling regulation was found to be cell- and effector-specific. As previously mentioned, this multiplicity of regulatory mechanisms argues against the possibility of developing a single universal means of controlling analgesic tolerance. But, on the other hand, it could provide a novel strategy for the development of more specific, longer acting analgesics. Indeed, diversity could be an advantage if it were to allow directing the pharmacological stimuli towards those receptors that are specifically involved in analgesic responses and whose cellular location and/or effector association would make them more resistant to progressive waning of signalling efficacy. In this sense it might be helpful to think of receptors not as isolated membrane proteins but as part of signalling complexes containing a combination of G proteins [14,92], effectors [93], scaffolding [94] and/or regulatory proteins whose identity is determined by the cell type and compartment in which receptors are expressed [95]. Within complexes formed in different cells, structural restrictions imposed by distinct interaction partners may force receptors and/or effectors into conformations which need not be equally recognized by regulatory proteins [5,96]. It would therefore be conceivable that development of ligands capable of specifically activating signalling complexes with the least capacity to trigger the regulatory mechanisms underlying tolerance could result in more prolonged analgesic actions than those of currently available opioids. A schematic representation of this idea is shown in Figure 2.

## V. Concluding remarks

Initial mechanistic hypotheses concerning the molecular bases of opioid tolerance focused on homologous desensitization, viewing internalization as a key protective step for maintaining analgesic efficacy. Neuronal and *in vivo *studies tend to partially confirm this view, but also point to a greater level of complexity where post-endocytic sorting and multiplicity of regulatory mechanisms argue against a simple, universal strategy for reducing tolerance. Embracing this diversity through the production of biased ligands capable of favouring recycling or of directing pharmacological stimuli towards signalling complexes that are more resistant to functional desensitization could constitute novel strategies for rational design of longer acting opioid analgesics.

## List of abbreviations

(SNC-80): ((+)-4-[(alpha R)-alpha-((2S,5R)-4-allyl-2,5-dimethyl-1-piperazinyl)-3-methoxybenzyl]-N, N-diethyl-benzamide); (CFA): complete Freund's adjuvant; (DAMGO): [D-Ala(2), N-Me-Phe(4), Gly(5)-ol]-enkephalin; (DORs): delta opioid receptors; (DRG): dorsal root ganglion; (ERK1/2): extracellular signalregulated kinases; (GASP): G protein coupled receptor associated sorting protein; (GIRK): G protein activated inward rectifier K+; (GPCRs): G protein-coupled receptors; (LC): locus coeruleus; (MORs): mu opioid receptors; (AR-M1000390): N, NDiethyl- 4-(phenylpiperidin-4-ylidenemethyl) benzamide.

## Competing interests

The authors declare that they have no competing interests.

## Authors' contributions

KN conducted reference research, contributed discussion and figure concerning signalling complexes. GP conducted reference research and wrote the review. All authors read and approved the final manuscript.
